# Peroperative Cooling in Rhinoplasty: Does it Differ?

**DOI:** 10.1007/s00266-024-04105-y

**Published:** 2024-05-28

**Authors:** Goksel Turhal, Veysel Berber, Efe Isler, Sercan Gode

**Affiliations:** 1 Department of Otolaryngology, Ege University School of Medicine, Izmir, Turkey; 2 Department of Otolaryngology, Sarikamis State Hospital, 36500 Kars, Turkey

**Keywords:** Rhinoplasty, Cooling, Edema, Ecchymosis, Pain, Cold

## Abstract

The main causes of ecchymosis and edema are osteotomy (bone manipulation), dissection of subcutaneous tissue, and skin manipulation in the rhinoplasty procedure. Eyelid edema following surgery can potentially affect visual acuity, particularly during the initial twenty-four hours after the procedure. These may also delay the patient’s return to their normal social life therefore hampering their quality of life. Various surgical and medical methods have been reported to address these issues. This study aimed to compare the effects of using cold saline (0–4 °C) versus room temperature saline (20–25 °C) irrigation throughout the surgery on postoperative edema, ecchymosis, and pain. Fifty patients who underwent open-approach primary rhinoplasty between August 2022 and August 2023 at a tertiary academic center were included. Fifty patients were randomly divided into two groups depending on using cold saline (0–4 °C) (group 1) or room temperature saline (20–25 °C) (group 2) during surgical site irrigation. Patients were assessed for pain, edema, and bruising using a VAS (Visual Analog Scale) on the second and seventh postoperative days. Visual analog score (VAS) was used for subjective outcome analyses. Each patient scored the severity of their periorbital ecchymosis on day two and seven. Periorbital ecchymosis was also evaluated on the second and seventh postoperative days using the SPREE (Surgeon Periorbital Rating of Edema and Ecchymosis) scale. On the second postoperative day, the VAS pain score in group 1, where cold water was used, was found to be statistically and significantly different from the control group (group 2) (*p* < 0.05). However, there was no statistically significant difference between both groups when comparing the VAS pain scores on the seventh postoperative day. Regarding the VAS ecchymosis score on the seventh postoperative day, there was a statistically significant difference favoring group 1 (*p* < 0.05). The SPREE scale data also indicated that group 1 had significantly lower scores on the seventh day (*p* < 0.05). While the SPREE scores on the second day were lower in group 1 than in group 2, this difference did not reach statistical significance (*p* = 0.061). The findings from our study show that cold saline irrigation may contribute to intraoperative hemostasis by inducing local vasoconstriction. We observed that intraoperative bleeding decreased with the use of cold saline. This approach has the potential to improve patient satisfaction and overall quality of life by reducing postoperative ecchymosis without significantly increasing the cost of the surgical procedure.

*Level of Evidence III* This journal requires that authors assign a level of evidence to each article. For a full description of these Evidence-Based Medicine ratings, please refer to the Table of Contents or the online Instructions to Authors www.springer.com/00266.

## Introduction

Rhinoplasty is one of the most common facial plastic surgery procedures. Soft tissue manipulation and surgical trauma to the nasal skeleton leads to intraoperative bleeding, subsequently periorbital postoperative edema, and ecchymosis. The main causes of ecchymosis and edema are osteotomy (bone manipulation), dissection of subcutaneous tissue, and skin manipulation in the rhinoplasty procedure [1–3]. Eyelid edema (swelling) following surgery can potentially affect visual acuity, particularly during the initial twenty-four hours after the procedure. These may also delay the patient’s return to their normal social life therefore hampering their quality of life. While a cautious surgical approach and meticulous dissection can help reduce this issue, it may not always be possible to entirely prevent it [4].

Various surgical and medical methods have been reported to address these issues. Currently, the primary surgical guidelines for minimally invasive rhinoplasty surgery emphasize meticulous dissection of the SMAS subplane (superficial musculoaponeurotic system) and avoiding harmful manipulations [1]. In addition to surgical techniques, different medical methods have been explored. Corticosteroids are effective in reducing edema and ecchymosis in the first few weeks after rhinoplasty. Some other approaches include inducing intraoperative hypotension and cooling the patient during surgery to reduce intraoperative bleeding. Additionally, the effects of mild hypocapnia, which causes vasoconstriction, on periorbital ecchymosis were also investigated [5]. This study aimed to compare the effects of using cold saline (0–4 °C) versus room temperature saline (20–25 °C) irrigation throughout the surgery on postoperative edema, ecchymosis, and pain.

## Materials and Methods

The study was designed as a randomized controlled prospective study. All procedures performed in the study were in concordance with the ethical standards of the institutional and/or national research committee and the Declaration of Helsinki in 1964 and its subsequent amendments or comparable ethical standards. Informed consent was obtained for surgery and photography, including the permission for publication of all patients who were included in the study. Fifty patients who underwent open-approach primary rhinoplasty between August 2022 and August 2023 at a tertiary academic center were included. Patients with a history of cancer, diabetes mellitus, rheumatologic and cardiovascular diseases were excluded.

All surgeries were performed by the same surgeon using the open-approach technique under general anesthesia. Anesthesia was induced with intravenous propofol 2 mg/kg and rocuronium 0.6 mg/kg and maintained with 2–3 vol% sevoflurane in oxygen in air adjusted to keep entropy values between 40 and 60. Remifentanil was initially administered at a rate of 0.1 g/kg/min before induction and infused at 0.05–0.15 g/kg/min to keep hemodynamic variables suitable to induce hypotension. Six cc lidocaine and 1:10000 adrenaline (1:1) injections were applied to the same locations and in equal doses before the operation. After the transcolumellar inverted V incision, skin soft tissue envelope was elevated through the sub-SMAS plane. The procedure then included septoplasty, dorsum reduction, and alar trimming, and concluded with medial oblique and lateral osteotomies. No powered instrument was used to achieve comparable results. Silicon nasal stents (Doyle 2; Medtronic Xomed F1, Ankara, Turkey) and external nasal splints (Rhinofix; Rinomed Tibbi Urunler, Istanbul, Turkey) were routinely used in postoperative care. One week of head elevation and 24 hours of postoperative ice packs were suggested. Postoperatively patients were prescribed oral amoxicillin-clavulanic acid and acetaminophene one week. Non-steroid anti-inflammatory drugs were avoided to abstain from their disruptive effects on platelet function.

**Fig 1:**
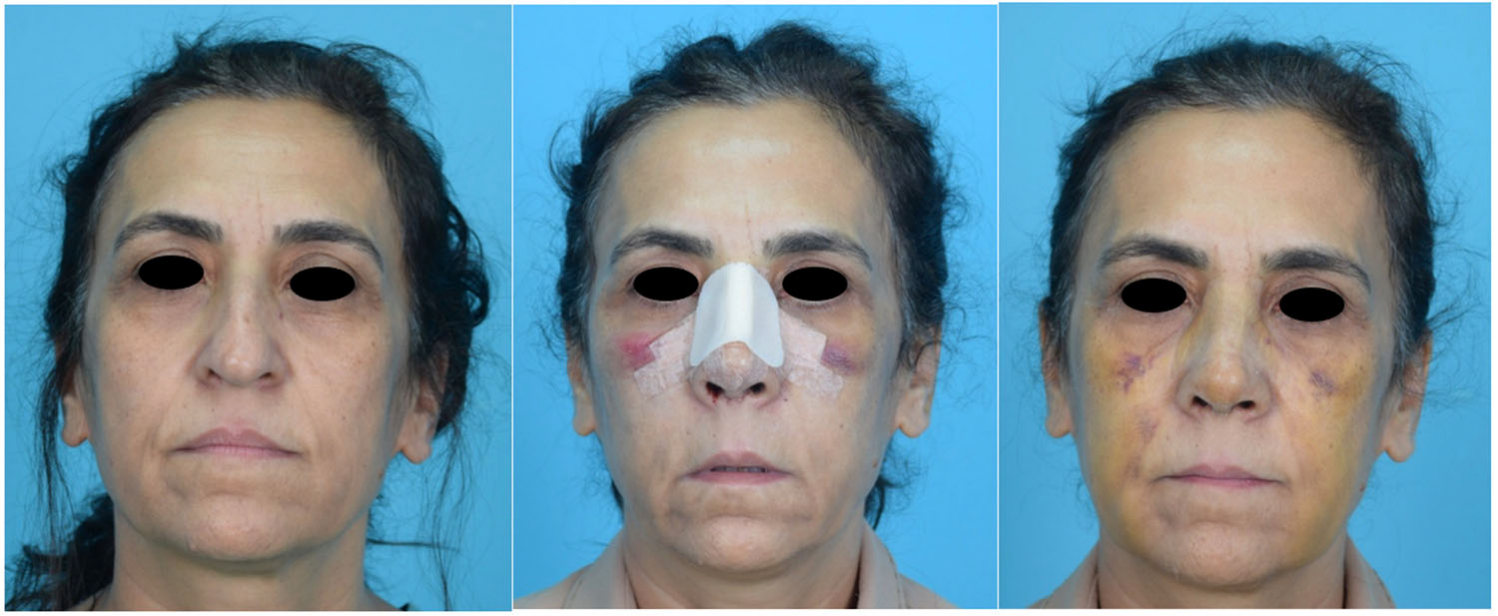
Group 1 (Control) patient preoperative, second day and seventh day images

**Fig 2:**
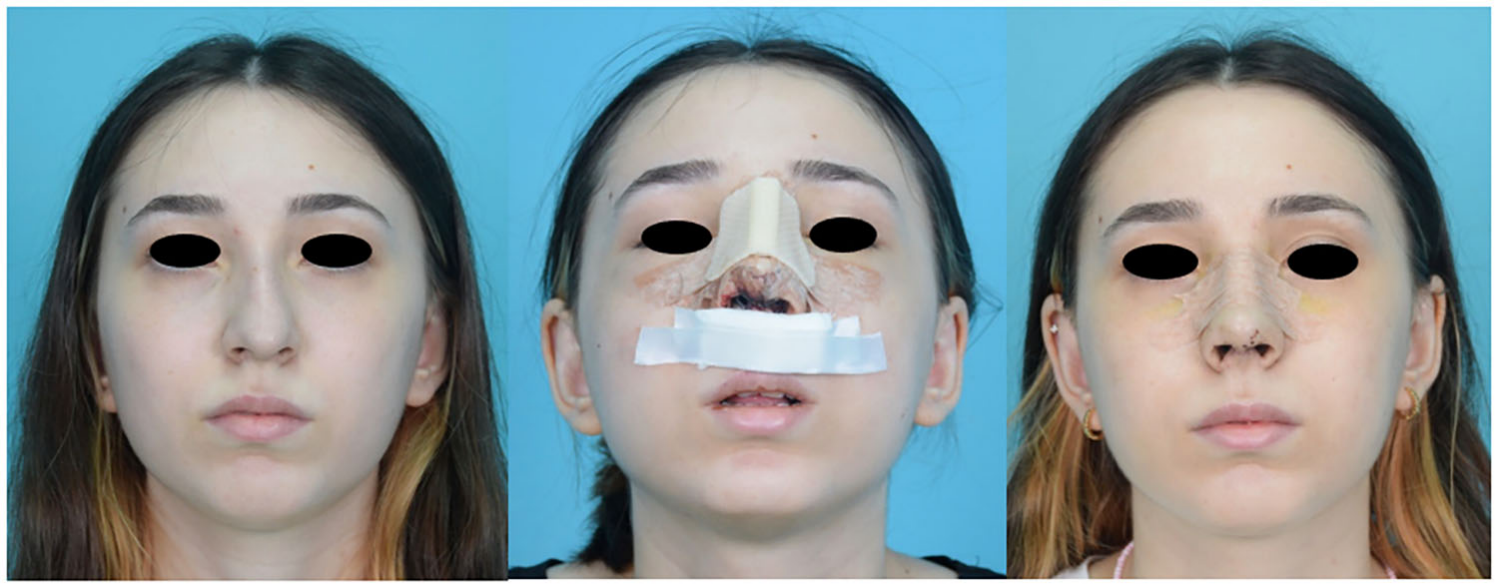
Group 2 (Cold water) patient preoperative, second day and seventh day images

Fifty patients were randomly divided into two groups depending on using cold saline (0–4 °C) (group 1) or room temperature saline (20–25 °C) (group 2) during surgical site irrigation. In group 1 the cold saline was replaced every 15 minutes to keep the irrigation fluid at the desired temperature. In both groups irrigation with saline was continuously used during incision, elevation of the skin flaps, following osteotomies, and suturation. Patients were assessed for pain, edema, and bruising using a VAS (Visual Analog Scale) on the second and seventh postoperative days. Visual analog score (VAS) was used for subjective outcome analyses. Each patient scored the severity of their periorbital ecchymosis on day two and sever. They were asked to score between 0 and 10. 0 was the best, whereas 10 was the worst outcome for ecchymosis. Periorbital ecchymosis was also evaluated on the second and seventh postoperative days using the SPREE (Surgeon Periorbital Rating of Edema and Ecchymosis) scale by an experienced surgeon, based on standardized photographs taken in the photography studio of the otolaryngology department with same camera settings and lighting conditions [6]. The patients were also stratified according to their Fitzpatrick skin types for further subgroup analysis. Each patient stood 2 meters away from the camera, and the visual axis was parallel to the floor of the room for the frontal, three-quarter, and lateral views. The camera head was horizontal to the lens of the camera (Nikon f2.8 105-mm macro lens, Nikon, Japan). A single camera-mounted flash was used with two synchronized studio flashes that were positioned at 45° from the subject-camera axis behind the photographer. No other light source was used. Patients were seated in a fixed position and asked to gaze directly at the fixed points for different views. Standard pictures were obtained: the eyes were fully open with a direct gaze, and the lips were closed with no smile (Figs. 1 and 2).

**Fig 3:**
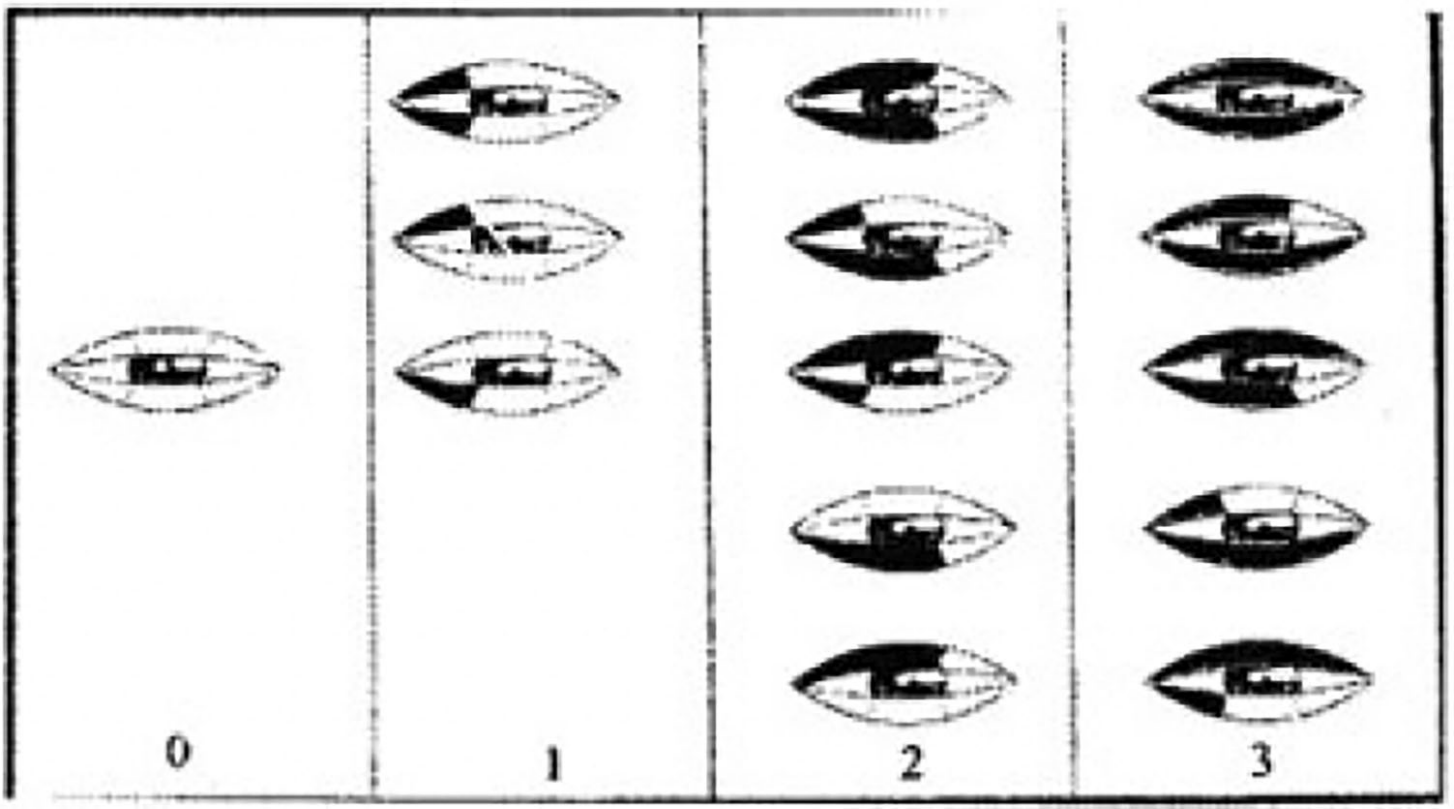
Surgeon periorbital rating of ecchymosis questionnaire

The grading system for evaluating periorbital ecchymosis is as follows (Fig. 3):

- 0 points: None (no ecchymosis).
- 1 point: Medial (ecchymosis limited to the medial area).
- 2 points: Extending to the pupil (ecchymosis reaching up to the pupil).
- 3 points: Extending past the pupil (ecchymosis extending beyond the pupil).
- 4 points: Extending to the lateral canthus (ecchymosis extending to the outer corner of the eye, known as the lateral canthus).

This grading system allows for the assessment and classification of the severity and extent of periorbital ecchymosis, with higher point values indicating more extensive and severe bruising around the eye.

**Fig 4:**
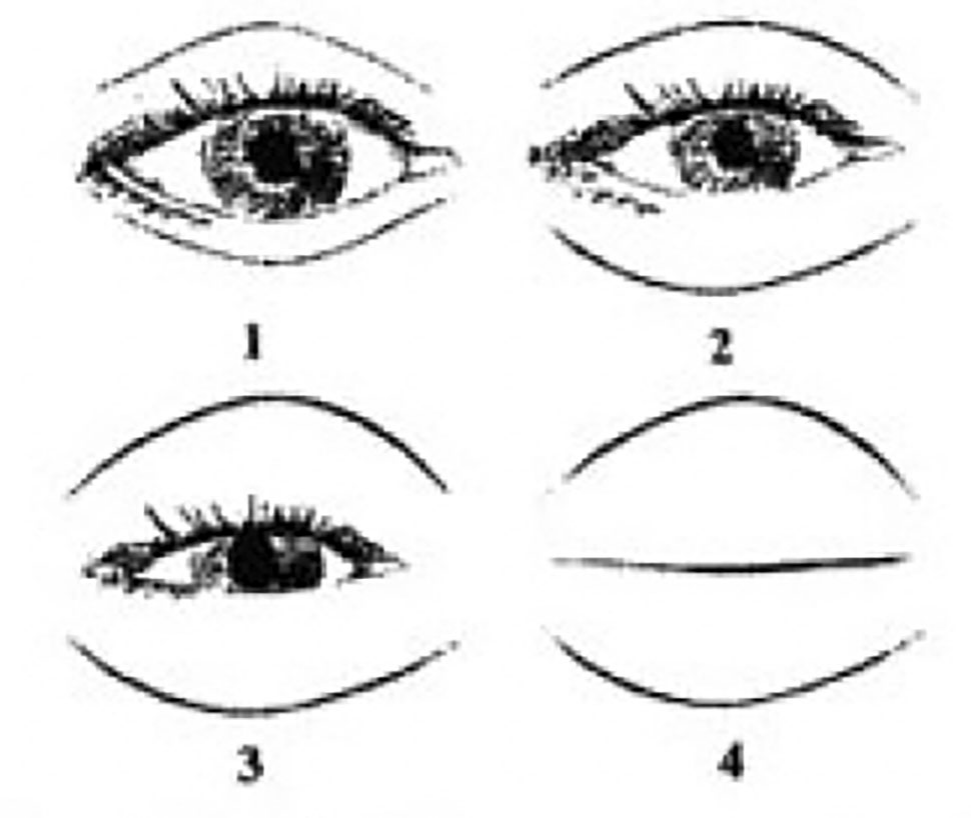
Surgeon periorbital rating of edema questionnaire

The grading system for evaluating eyelid edema consists of the following points (Fig. 4):

- 0 points: None (no edema)
- 1 point: Minimal (minimal edema)
- 2 points: Extending onto the iris (edema extending onto the iris)
- 3 points: Covering the iris (edema covering the iris)
- 4 points: Massive edema with the eyelid swollen shut (severe edema, completely obstructing the eye)

This grading system allows for the assessment and classification of the severity of eyelid edema based on its extent and impact on the eye. The higher the point value, the more severe the edema is considered to be.

## Statistical Analysis

Statistical analyses were conducted using the SPSS® 25.0 (Statistical Package for Social Science) program, developed by SPSS Inc. in Chicago, IL, USA. The Shapiro–Wilk test was used for determining the distribution pattern of the data. Within-group comparisons of variables were performed using ANOVA, while between-group comparisons of variables were assessed using the Levene test. All data were expressed as “mean ± standard deviation”.

## Results

A total of 50 patients (20 (40%) men and 30 (60%) women) were included in the study (Table 1). The mean age of the patients was 28.78 ± 10.18 (range 18–55) years. Two (4%) patients had Fitzpatrick skin type 1, 13 (26%) had type 2, 19 (38%) had type 3, and 16 (32%) had type 4 (Table 2). None of the patients had Fitzpatrick type 5 or 6 skin. The average duration of the surgical procedure was 122.4 ±  14.8 minutes, with a range of 92–145 minutes.

**Table 1:** Distribution of participants by gender

Gender	Group 1 (Control)	Group 2 (Cold Water)	Total
Female	16 (32%)	14 (28%)	30 (60%)
Male	9 (18%)	11 (22%)	20 (40%)

**Table 2:** Fitzpatrick skin types by gender

Fitzpatrick skin type	1	2	3	4
Male	0	0	9 (18%)	11 (22)
Female	2 (4%)	13 (26%)	10 (20%)	5 (10%)

Mean second and seventh day VAS edema, second and seventh day VAS bruising, second and seventh day VAS pain, second and seventh day SPREE scores are present in tables 3, 4 and 5. Second day mean VAS pain scores (5.52 ± 1.96 vs 3.36 ± 2.97) and seventh day VAS bruising scores (4.20 ± 2.98 vs 2.16 ± 2.41) were significantly lower in the cold water group (*p* = 0.004 and *p* = 0.011). Regarding SPREE scores, the seventh day values of the cold water group was significantly lower (1.08 ± 0.76 vs 0.52 ± 0.77) in the cold water group (*p* = 0.013). The second day SPREE score was also lower in the cold water group (2.92 ±  0.81 vs 2.40 ± 1.08); however, this did not reach statistical significance (*p* = 0.060).

**Table 3:** SPREE scores on the second day

Second day SPREE score	Group 1 (Control)	Group 2 (Cold water)	Total
0	0	0	0
1	1	6	7
2	6	8	14
3	12	6	18
4	6	5	11

SPREE: Surgeon periorbital rating of edema and ecchymosis

**Table 4:** SPREE scores on the seventh day

Seventh day SPREE score	Group 1 (Control)	Group 2 (Cold water)	Total
0	6	15	21
1	11	8	19
2	8	1	9
3	0	1	1
4	0	0	0

SPREE: Surgeon periorbital rating of edema and ecchymosis

**Table 5:** Mean VAS and SPREE scores

	Group	Mean score ± standard deviation	*P* value
VAS second day Edema	Group 1	5.00+2.06	*p* = 0.348
	Group 2	4.36+2.67	
VAS second day Bruising	Group 1	4.52+2.31	*p* = 0.758
	Group 2	4.72+2.24	
VAS second day pain	Group 1	5.52+1.96	*p* = 0.004
	Group 2	3.36+2.97	
VAS seventh day edema	Group 1	4.52+2.87	*p* = 0.071
	Group 2	3.08+2.62	
VAS seventh day bruising	Group 1	4.20+2.98	*p* = 0.011
	Group 2	2.16+2.41	
VAS seventh day pain	Group 1	3.56+2.56	*p* = 0.268
	Group 2	2.72+2.73	
SPREE second day	Group 1	2.9210.81	*p* = 0.060
	Group 2	2.40+1.08	
SPREE seventh day	Group 1	1.08+JD.76	*p* = 0.013
	Group 2	0.52+0.77	

The study found no significant difference in the influence of the Fitzpatrick skin type on postoperative VAS bruising, edema, pain, and SPREE scores in both groups (Table 6). Additionally, no significant difference was observed between gender and VAS bruising, VAS edema, VAS pain, and SPREE scores on the second and seventh postoperative days.

**Table 6:** Subgroup analysis of VAS and SPREE scores according to Fitzpatrick skin type

VAS second day ecchymosis	0.376
VAS second day bruising	0.719
VAS second day pain	0.722
VAS seventh day ecchymosis	0.334
VAS seventh day bruising	0.185
VAS seventh day pain	0.132
SPREE second day	0.114
SPREE seventh day	0.902

## Discussion

Septorhinoplasty is associated with postoperative periorbital ecchymosis and edema, significantly affecting patients' postoperative recovery [7–10]. Edema and ecchymosis in the periorbital region are frequent outcomes of the trauma inflicted on both soft and bony tissues during rhinoplasty. Following the operation, ecchymosis of the eyelids typically intensifies during the first two days. However, complete recovery from ecchymosis is usually observed around the ninth day, and patients typically resume their normal social activities [11].

Various methods have been used to reduce postoperative edema and ecchymosis, including using steroids, decongestants, herbal supplements, vibration therapy, nasal packing, and cold applications [4, 8, 9]. During septorhinoplasty, the surgical area is intermittently irrigated with sterile saline to remove bone fragments, reduce soft tissue trauma, and improve visibility. However, the surgery inherently causes bone and soft tissue trauma, leading to inflammation and disruption of lymphatic and venous drainage, resulting in ecchymosis. To reduce ecchymosis, some studies have explored interventions in the coagulation cascade, intraoperative vasoconstriction, and hypotension. Ice application has been widely used after maxillofacial and septorhinoplasty surgeries due to its ability to disrupt blood circulation by vasoconstriction, lymphatic drainage, and provide analgesic effects [11–14].

In a study, combined effectiveness of intraoperative cold saline-soaked gauze and corticosteroids was examined to reduce postoperative eyelid edema and periorbital ecchymosis. The study found that these interventions led to significant reductions in eyelid edema and periorbital ecchymosis on the following postoperative days: 1, 3, 5, and 7 [15]. Similarly, in our study, patients who received cold saline irrigation reported lower VAS pain scores on the second postoperative day. This suggests that the use of cold saline can lead to reduced postoperative pain, potentiating patient comfort during the early recovery period.

Although steroid use reduces postoperative ecchymosis and edema, the effectiveness of steroids in this context has been a subject of debate, and there is no consensus regarding the specific drug or dosage regimen to achieve consistent results. Gurlek et al suggested that steroids were not effective in reducing postoperative edema and ecchymosis [4]. However, in a separate study, a high dose of methylprednisolone was effective in preventing and reducing periorbital edema and ecchymosis. This highlights that the efficacy of steroids may depend on the specific steroid used and the dosage employed [7]. In a meta-analysis, it was reported that postoperative and long-term steroid use outperformed single-dose administration in reducing edema and ecchymosis [16]. To achieve the desired efficacy with steroids, a high dose must be administered for an extended duration, typically up to one week postoperatively. However, this approach may not be suitable for all patients due to potential side effects and individual patient characteristics.

Controlled hypotension might be beneficial when used either alone or in combination with local agents for managing postoperative ecchymosis and edema [17, 18]. It may be a more reasonable option, especially for younger patients, as it avoids the potential risks and side effects associated with various medications. Numerous drugs have been studied to achieve controlled hypotension, which is typically defined as reducing systolic blood pressure to 80–90 mmHg, reducing mean arterial pressure (MAP) to 50–65 mmHg, or achieving a 30% reduction in baseline MAP. Maintaining a MAP of 50–60 mmHg is considered optimal [19, 20]. However, attempting to maintain such low blood pressure levels may lead to undesirable and unexpected hemodynamic changes, including hypotension, reflex tachycardia, systemic hypertension, arterial hypertension, arrhythmias, and pulmonary hypertension [21, 22].

Various pharmacological agents have been described in the literature to control intraoperative bleeding in rhinoplasty. Tranexamic acid is a pharmacological agent used to reduce intraoperative bleeding. It functions as an antifibrinolytic and is commonly used in both cardiac and non-cardiac surgeries to reduce intraoperative bleeding and the need for blood transfusions [23]. Eftekharian et al investigated the use of preoperative oral tranexamic acid in rhinoplasty procedures and found a significant reduction in intraoperative bleeding with no reported side effects [24]. Some studies suggest interventions that cause intraoperative vasoconstriction, such as mild hypocarbia, might reduce bleeding and postoperative ecchymosis [5].

The findings from our study show that cold saline irrigation may contribute to intraoperative hemostasis by inducing local vasoconstriction. In our study, we observed that intraoperative bleeding decreased with the use of cold saline. This reduction is likely due to the local vasoconstriction induced by the cold saline, which helps mitigate blood vessel dilation and the release of local mediators that contribute to bleeding. This approach has the potential to improve patient satisfaction and overall quality of life by reducing postoperative ecchymosis without significantly increasing the cost of the surgical procedure.

Postoperative systemic side effects may arise from the frequent use of pharmacological methods to reduce edema and ecchymosis following rhinoplasty. In the case of cold saline surgical site irrigation examined in our study, no systemic or hemodynamic effects were observed as no systemic medication was administered to the patients. This makes the use of intraoperative cold saline suitable for every patient. Furthermore, since no additional medications are given, the risk of complications related to the patient's comorbid conditions is minimized. This method is easy to apply, does not require additional costs, and is thought to have no negative effects on the patient. Our objective was to create a surgical field with minimal bleeding, reduced edema, and ecchymosis in the postoperative period. Periorbital ecchymosis, as assessed by the SPREE scale by an independent surgeon, showed a positive association with perioperative cold water irrigation on both the second and seventh postoperative days. This indicates that irrigation with cold water during surgery may have a beneficial impact on reducing ecchymosis. The study also investigated the influence of Fitzpatrick skin types on the outcomes, considering that ecchymosis may be less visible in individuals with darker skin tones. However, the study did not find any significant effect of Fitzpatrick skin types on the outcomes. It is important to note that the patient group lacked individuals with Fitzpatrick types 5 and 6. Future research should include a larger and more diverse patient population to unveil the postoperative ecchymosis and skin type relation.

## Conclusion

In conclusion, this study highlights the potential benefits of using cold saline irrigation during rhinoplasty. It has shown promise in terms of reducing postoperative pain and ecchymosis. Additionally, investigation of economic aspects and patient-reported outcomes may provide valuable information regarding the overall impact of this technique on patient satisfaction and quality of life.
